# A Comparison of 14 *Erythrobacter* Genomes Provides Insights into the Genomic Divergence and Scattered Distribution of Phototrophs

**DOI:** 10.3389/fmicb.2016.00984

**Published:** 2016-06-24

**Authors:** Qiang Zheng, Wenxin Lin, Yanting Liu, Chang Chen, Nianzhi Jiao

**Affiliations:** ^1^State Key Laboratory of Marine Environmental Science, Institute of Marine Microbes and Ecospheres, Xiamen UniversityXiamen, China; ^2^CAS Key Laboratory of Tropical Marine Bio-resources and Ecology, South China Sea Institute of Oceanology, Chinese Academy of SciencesGuangzhou, China; ^3^Xisha Deep Sea Marine Environment Observation and Research Station, South China Sea Institute of Oceanology, Chinese Academy of SciencesSansha, China

**Keywords:** *Erythrobacter*, integrative and conjunctive element, photosynthesis gene cluster, aerobic anoxygenic phototrophic bacteria, comparative genomics

## Abstract

Aerobic anoxygenic phototrophic bacteria (AAPB) are bacteriochlorophyll *a* (Bchl *a*)-containing microbial functional population. *Erythrobacter* is the first genus that was identified to contain AAPB species. Here, we compared 14 *Erythrobacter* genomes: seven phototrophic strains and seven non- phototrophic strains. Interestingly, AAPB strains are scattered in this genus based on their phylogenetic relationships. All 14 strains could be clustered into three groups based on phylo-genomic analysis, average genomic nucleotide identity and the phylogeny of signature genes (16S rRNA and *virB4* genes). The AAPB strains were distributed in three groups, and gain and loss of phototrophic genes co-occurred in the evolutionary history of the genus *Erythrobacter*. The organization and structure of photosynthesis gene clusters (PGCs) in seven AAPB genomes displayed high synteny of major regions except for few insertions. The 14 *Erythrobacter* genomes had a large range of genome sizes, from 2.72 to 3.60 M, and the sizes of the core and pan- genomes were 1231 and 8170 orthologous clusters, respectively. Integrative and conjugative elements (ICEs) were frequently identified in genomes we studied, which might play significant roles in shaping or contributing to the pan-genome of *Erythrobacter*. Our findings suggest the ongoing evolutionary divergence of *Erythrobacter* genomes and the scattered distribution characteristic of PGC.

## Introduction

Aerobic anoxygenic photoheterotrophic bacteria (AAPB) are bacteriochlorophyll *a* (Bchl *a*)-containing and obligate aerobic bacteria, and they are widely distributed in the euphotic zone of the ocean (Kolber et al., 2001; Yurkov and Csotonyi, 2009). These phototrophic microorganisms account for 1–15% of the total bacteria in the upper ocean, and the BChl *a*-based phototrophy could reduce respiratory organic carbon consumption of ~2.4–5.4% of marine primary productions (Jiao et al., 2010; Ritchie and Johnson, 2012; Ferrera et al., 2014; Stegman et al., 2014). Thus, AAPB could potentially play significant roles in carbon and energy cycling in the ocean (Kolber et al., 2001; Jiao et al., 2007; Koblížek et al., 2007).

Currently, the known marine AAPB isolates are classified into *Proteobacteria*, including *Alpha-* and *Gammaproteobacteria*. *Alphaproteobacterial* AAPB mainly comprises the *Roseobacter* clade (e.g., genera *Roseobacter, Dinoroseobacter, Roseivivax, Roseovairus*, and *Roseibacterium*) and the Eryth-Citro clade, including the genera *Erythrobacter* and *Citromicrobium* (Béjà et al., 2002; Yutin et al., 2007; Zheng et al., 2011). Most cultured marine *Gammaproteobacterial* AAPB belong to the clade NOR5/OM60 (Cho et al., 2007; Fuchs et al., 2007; Spring et al., 2009).

AAPB possess a highly conserved photosynthesis gene cluster (PGC) including *bch, crt, puf*, *puh*, and some regulatory genes (Blankenship, 1992; Beatty, 1995; Swingley et al., 2009; Zheng et al., 2011). For the evolutionary history of AAPB, both gain and loss of PGC were detected as revealed by comparison of AAPB and closely related non-AAPB genomes. *Gemmatimonas* sp. AP64, which belongs to phylum *Gemmatinonadetes*, might obtain its PGC from purple phototrophic bacteria (Alplaproteobacteria) by horizontal gene transfer (Zeng et al., 2014). *Citromicrobium* sp. JLT1363, which is classified into Alpha-IV subclade, lost the PGC from the long-term evolutionary history and became a completely heterotrophic bacterium (Zheng et al., 2012). Previous studies have shown that the AAPB in the Eryth-Citro clade contain unique carotenoid biosynthetic pathways and pigments compared with the *Roseobacter* clade (Koblížek et al., 2003; Zheng et al., 2011, 2013). The structure of the photosynthesis gene cluster (PGC) in the Eryth-Citro clade was the shortest and simplest among all known AAPB, and no light-harvesting complex II (LH II) genes were found in their genomes (Zheng et al., 2011, 2013). The obligate aerobic characteristics and unique PGC structure suggest that these AAPB in the Eryth-Citro diverged long time ago with AAPB belonging to *Roseobacter* clade (Zheng et al., 2012, 2013, 2014). However, few studies have been focused on the evolution of AAPB in *Erythrobacter* genus to date, although a great number of strains have been sequenced in this genus.

The genus *Erythrobacter* was established following the isolation and identification in 1982 of the first AAPB strain *Erythrobacter longus* DSM 6997 (Shiba and Simidu, 1982). Later, the second AAPB species belonging to *Erythrobacter* genus, *E. litoralis* DSM 8509, was identified by Yurkov et al. (1994). Since then, a variety of strains belonging to this genus have been recognized from diverse habitats (Koblížek et al., 2003; Zheng et al., 2014; Lei et al., 2015; Zhuang et al., 2015), and some of them lack Bchl *a* (Anderson et al., 2009; Oh et al., 2009; Wei et al., 2013). By the end of 2015, 18 species and dozens of strains had been isolated and identified in the genus *Erythrobacter* (Tonon et al., 2014; Zheng et al., 2014; Lei et al., 2015; Zhuang et al., 2015). Interestingly, photoheterotrophic bacteria did not cluster together within one genus based on their phylogenetic relationship. The aim of this study is trying to address (1) the evolutionary divergence of their genomes and (2) the distribution pattern and evolution of photosynthesis gene cluster in the genus *Erythrobacter*.

## Materials and methods

### Bacterial strains

Fourteen *Erythrobacter* spp. strains were used for bioinformatic analyses. Five of these were sequenced by our lab: *Erythrobacter longus* DSM 6997, *Erythrobacter litoralis* DSM 8509, *Erythrobacter* sp. JL475, *Erythrobacter* sp. YT30, and *Erythrobacter* sp. AP23. *Erythrobacter longus* DSM 6997 and *Erythrobacter litoralis* DSM 8509 were purchased from the DSMZ culture collections. Strains JL475, YT30, and AP23 were isolated from the South China Sea using extinction dilution method on rich organic medium (Yurkov et al., 1999) and maintained in the laboratory.

The other nine *Erythrobacter* genomes were collected from the National Center for Biotechnology Information (NCBI), and their GenBank accession numbers follow: *Erythrobacter* sp. NAP1 (AAMW00000000; Koblížek et al., 2011), *Erythrobacter marinus* HWDM-33 (LBHU00000000; Jung et al., 2012), *Erythrobacter litoralis* HTCC2594 (NC007722; Oh et al., 2009), *Erythrobacter* sp. s21-N3 (CP011310; Zhuang et al., 2015), *Erythrobacter* sp. SD-21 (ABCG00000000; Anderson et al., 2009), *Erythrobacter gangjinensis* K7-2 (LBHC00000000; Lee et al., 2010), *Erythrobacter vulgaris* O1 (CCSI00000000; Yaakop et al., 2015), *Erythrobacter* citreus LAMA 915 (JYNE00000000), and *Erythrobacter* sp. KA37 (LBHB00000000; Lei et al., 2015).

### Genome sequencing, assembly, and annotation

Three draft genomes of strains DSM 6997, DSM 8509, and JL475 were obtained using Illumina HiSeq sequencing technology in Chinese National Human Genome Center at Shanghai. Two libraries with average sizes of 150 and 500 bp were constructed using the TruSeq TM DNA Library Prep Kit (Illumina, USA). Paired-end reads of an average length of 100 bp were assembled using Velvet software (V1.2.03) (Zerbino and Birney, 2008), and a total read size of ~2.5 Gbp for each strain was obtained.

The genomes of strains YT30 and AP23 were obtained using the Illumina MiSeq system in Shanghai Personal Biotechnology Limited Company. Paired-end reads of an average length of 250 bp were assembled using Velvet software (v2.8; Zerbino and Birney, 2008). The sequencing coverage was ~300X for strains AP23 and YT30.

The prediction and annotation of open reading frames (ORFs) were performed with Rapid Annotation using Subsystem Technology (RAST; Aziz et al., 2008). The rRNA and tRNA identification was performed with RNAmmer 1.2 software (Lagesen et al., 2007) and tRNAscan-SE (v1.21; Lowe and Eddy, 1997), respectively.

The genomic average nucleotide identity (ANI) was calculated with the JSpecies Web online service (http://jspecies.ribohost.com/jspeciesws; Richter and Rosselló-Móra, 2009).

### Core genome and pan-genome analyses

Orthologous clusters (OCs) were analyzed using OrthoMCL, and all protein sequences from the 14 genomes were grouped based on the sequence similarity (*E* < 10^−5^, >50% coverage; Li et al., 2003). All genes from fourteen *Erythrobacter* genomes were selected to calculate the core and pan-genomes. The sizes of core and pan-genomes were calculated based on the number of genomes involved in the analysis (Tettelin et al., 2005).

### Phylogenetic analysis

The sequences were aligned using Clustal X, and phylogenetic trees were constructed using the neighbor-joining and maximum likelihood algorithms of MEGA 6 software (Tamura et al., 2013). The phylogenetic trees were supported by bootstrap for the resampling test with 1000 and 100 replicates for the neighbor-joining and maximum likelihood algorithms, respectively.

### Accession numbers

The whole-genome sequences of strains DSM 6997, DSM8509, JL475, AP23, and YT30 are available under GenBank accession numbers JMIW00000000, JMIX00000000, JMIV00000000, LNBY00000000, and LMAF00000000, respectively.

## Results and discussion

### General features of the *Erythrobacter* strains

Fourteen strains isolated from diverse aquatic environments were used for comparative genome analyses (Table 1). Seven of them containing complete PGC were AAPB strains, including *Erythrobacter* sp. NAP1, *E. longus* DSM 6997, *E. litoralis* DSM 8509, and *Erythrobacter* sp. JL475, *Erythrobacter* sp. AP23, *Erythrobacter* sp. YT30 and *E. marinus* HWDM-33. The other seven strains containing no PGC were non-AAPB. All shared more than 94% 16S rRNA sequence similarity. However, the nucleotide identities among the seven *pufM* sequences were < 80%.

**Table 1:** **Genome information for the 14 strains**.

	**Strain**	**Acc. No**.	**Genome size (M)**	**Genome GC**	**Genes**	**Structural RNAs**	**PGC size (Kb)**	**PGC GC**	**Contigs**	**ICE type**	**Coverage**	**Isolation source**	**References**
Non-AAPB	*E. gangjinensis* K7-2	LBHC00000000	2.72	0.63	2648	43	–	–	8	–	–	Seawater of Gangjin Bay, South Korea	Lee et al., 2010
	*E. vulgaris* O1	CCSI00000000	2.86	0.62	2800	46	–	–	11	–	–	Malaysian beach	Yaakop et al., 2015
	*Erythrobacter* sp. KA37	LBHB00000000	2.89	0.58	2921	45	–	–	22	–	–	Mangrove sediment	Lei et al., 2015
	*Erythrobacter* sp. SD-21	ABCG00000000	2.97	0.62	2985	45	–	–	19	I	–	San Diego Bay	Anderson et al., 2009
	*Erythrobacter* sp. s21-N3	CP011310	3.01	0.58	2921	45	–	–	19	I, II	–	Deep sea sediment from the Atlantic Ocean	Zhuang et al., 2015
	*E. litoralis* HTCC2594	NC007722	3.05	0.63	3056	45	–	–	1	I, II, III-A	–	10 m in the Sargasso Sea	Oh et al., 2009
	*E. citreus* LAMA 915	JYNE00000000	3.09	0.64	2999	45	–	–	28	I	–	Deep sea water from the Atlantic Ocean	–
AAPB	*E. marinus* HWDM-33	LBHU00000000	2.84	0.59	2701	44	38.6	0.60	10	–	–	Seawater, Yellow Sea	Jung et al., 2012
	*Erythrobacter* sp. YT30	LMAF00000000	3.20	0.57	3081	43	38.1	0.59	6	–	299x	Seawater from the South China Sea	This study
	*E. litoralis* 8509	JMIX00000000	3.22	0.65	3052	44	38.9^*^	0.65	20	–	780x	Marine cyanobacterial mat	This study
	*Erythrobacter* sp. NAP1	AAMW00000000	3.27	0.60	3223	46	38.9	0.62	4	III-A	–	Seawater from the Atlantic Ocean	Koblížek et al., 2011
	*Erythrobacter* sp. JL475	JMIV00000000	3.27	0.62	3117	44	38.8	0.63	4	III-A/B	780x	Seawater from the South China Sea	This study
	*Erythrobacter* sp. AP23	LNBY00000000	3.40	0.63	3251	46	37.0	0.66	20	I, II	298x	Seawater from the South China Sea	This study
	*E. longus* 6997	JMIW00000000	3.60	0.57	3363	42	38.8	0.58	12	I, III-C	680x	Seaweed	This study

* Means two large inserted genes were removed from the PGC.

**Figure 1:**
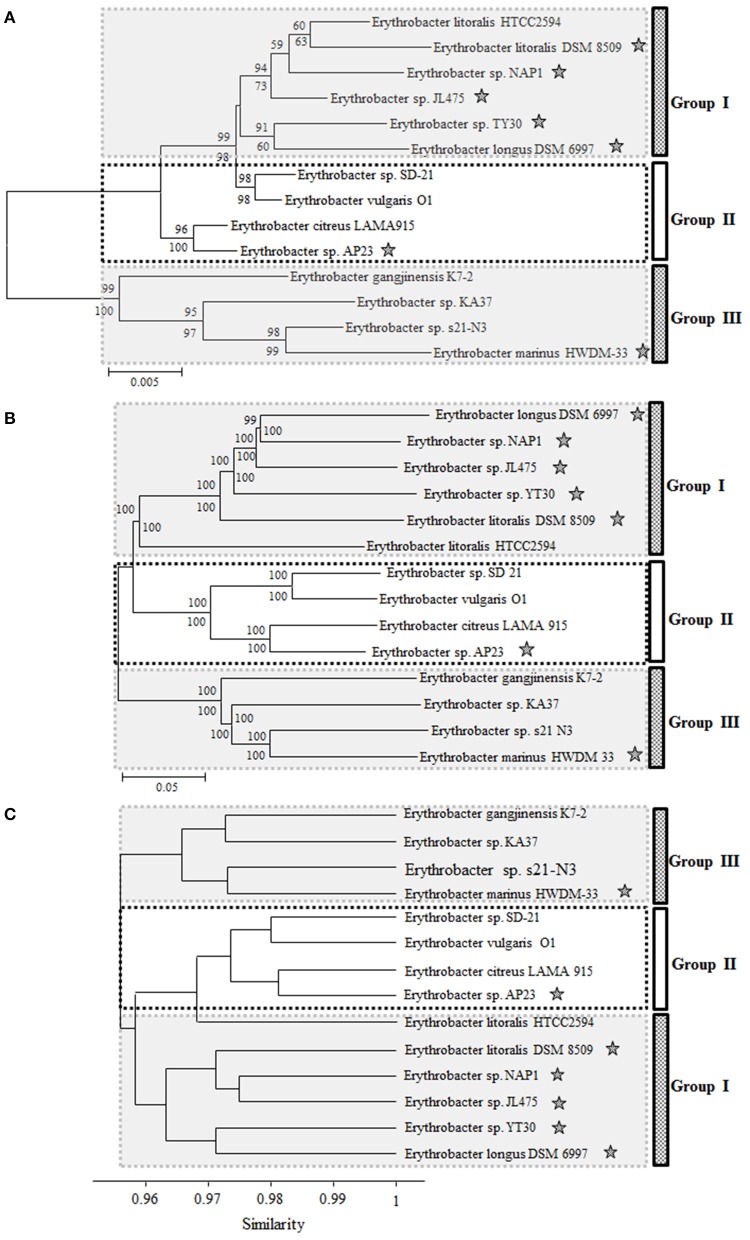
**Neighbor-joining phylogenetic trees based on the 16S rRNA gene (A) and concatenated amino acids sequences of 1167 universally conserved genes (B)**. Bootstrap percentages from both neighbor-joining (above nodes) and maximum likelihood (below nodes) are shown. **(C)** Cluster analysis based on the ANI from pairwise genome comparison. ^⋆^Represents the strain containing PGC.

From the phylogenetic trees based on the 16S rRNA gene, 14 *Erythrobacter* strains could be clustered into three groups: strains DSM 6997, NAP1, JL475, DSM 8509, YT30, and HTCC2594 formed one group (Group I), strains SD-21, O1, LAMA 915, and AP23 formed a second group (Group II), and the other four strains formed a third group (Group III; Figure 1A). The AAPB strains were scattered in three groups. The 16S rRNA sequence identities within each group (I and II) were more than 97 and 98%, respectively. The strains belonging to Group III showed much lower identities than the other two groups, ranging from 94 to 97%. Seven AAPB strains were grouped into three clades. The scattered distribution pattern of phototrophs was also found in *Rosoebacter* clade (Wagner-Döbler and Biebl, 2006; Koblížek et al., 2013). That indicates the evolution and distribution pattern of PGCs might be prevalent in Alphaproteobacteria.

### Genomic information for the *Erythrobacter* strains

The genomic size varied from 2.72 M (*E. gangjinensis* K7-2) to 3.60 M (*E. longus* 6997), and the total GC content ranged from 57 to 65% (Table 1). The number of genes is consistent with the genomic size. The number of structural RNAs ranged from 42 to 46. The average genome coverage for newly sequenced five strains, JL475, DSM 6997, DSM 8509, YT30, and AP23 were 780X, 680X, 780X, 299X, and 298X, respectively (Table 1).

The phylogenetic tree based on the concatenated amino acids sequences of 1167 universally conserved genes among these 14 investigated *Erythrobacter* genomes was consistent with 16S rRNA phylogeny and also formed three groups (I, II, and III) (Figure 1B). The genomic sizes of AAPB in Group I (3.20~3.60 M vs. 3.05 M) and II (3.40 M vs. 2.86~3.09 M) were significantly larger than non-AAPB in the same groups. All four strains in Group III (avg. ~2.86 M) showed relatively small genomic sizes compared with the other two groups (avg. ~3.19 M).

### The genomic average nucleotide identity

The Average Nucleotide Identity (ANI) shared between *Erythrobacter* genome pairs ranged from 68.78 to 81.34% (Table S1). Taking into account the proposed cut-off of the ANI between genome pairs for a species boundary of 95–96% (Richter and Rosselló-Móra, 2009), all analyzed 14 *Erythrobacter* strains were separated species; however, some of them shared high 16S rRNA sequence identities. The low genomic percentage (ranging from 33.60 to 70.81%) involved in pairwise comparisons indicated that they diverged a long time ago.

The genome pairs in each group showed relatively high ANI and genomic percentages involved in pairwise comparisons. The genome pairs in Group III shared 72.77–74.58% ANI, and the genomic percentages involved in pairwise comparisons ranged from 48.05 to 59.81% (Table S1). While the ANI values between genome pairs (one from Group III and the other from Group I or II) were 68.78–70.40% and 70.16–70.96%, respectively, the genomic percentages involved in pairwise comparisons were 36.31–41.32% and 37.05–44.42%, respectively (Table S1).

The ANI and genomic percentages involved in pairwise comparisons (Group II vs. Group I or III; Group I vs. Group II or III) had similar characteristics to Group III vs. Group I or II. In consistency with the 16S rRNA phylogenetic tree, strain HTCC2594 was clustered into Group I in the whole genome tree. However, strain HTCC2594 shared a higher ANI and involved a larger genomic percentage according to the pairwise comparison with the genomes in Group II (Figure 1C).

### The pan- and core genomes of the *Erythrobacter* strains

The *Erythrobacter* pan-genome for 14 sequence strains comprised 8170 predicted orthologous clusters (OCs), and the core genome contained 1231 OCs (Figure S1). The cumulative length of all core genes was approximately 1.20 Mbp, which covered only 33–45% of the genome content. The flexible genome comprises 6939 OCs including 3815 unique OCs and 3124 shared by more than one strain but not all strains. The number of genes for the core genome appeared to reach a plateau, whereas the genes for the pan-genome increased with genome number (Figure S1).

**Figure 2:**
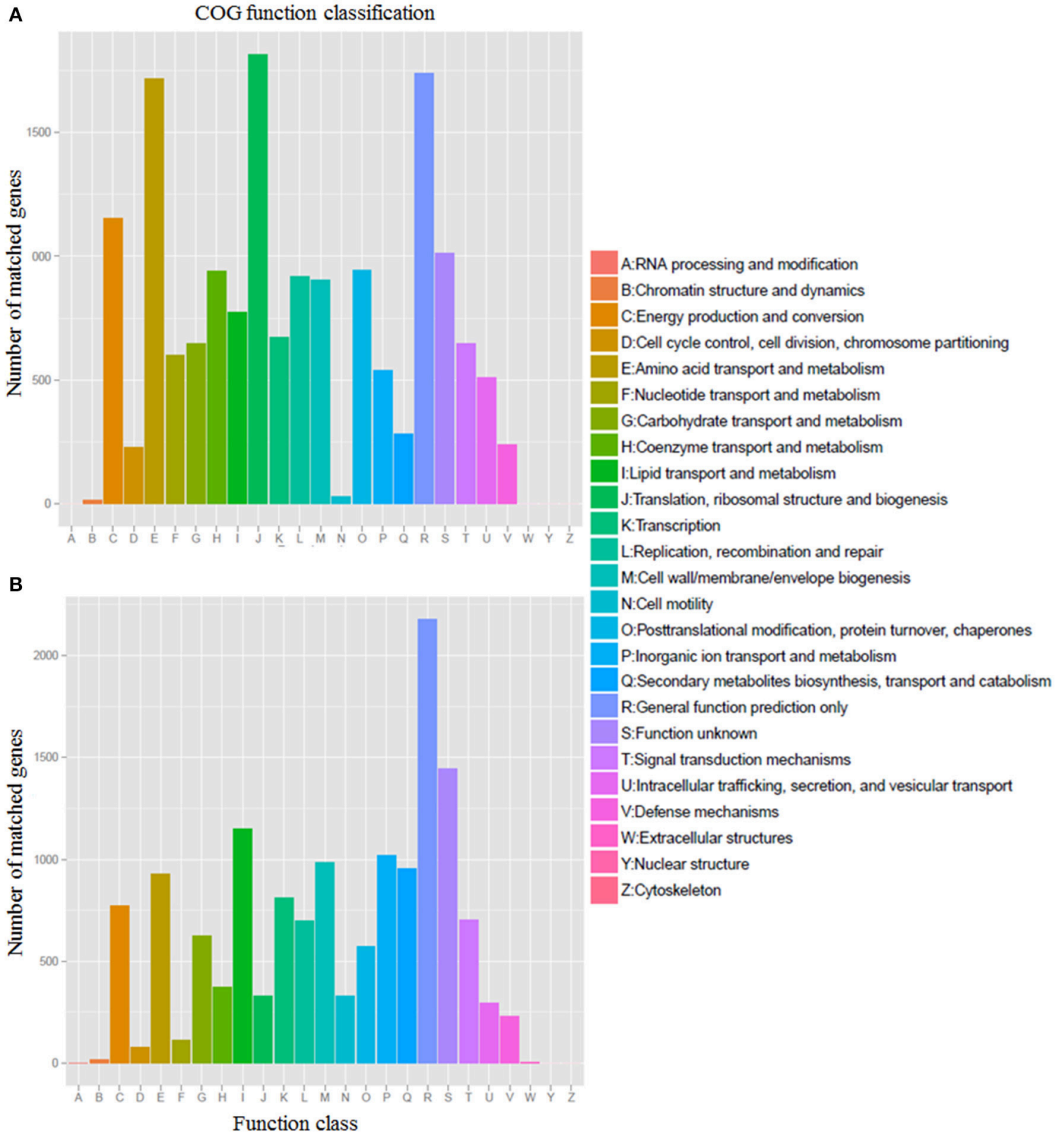
**COG function classification of core (A) and flexible (B) genes from 14 *Erythrobacter genomes***.

The core genome is mainly involved in central metabolism and housekeeping functions, from the Glycolysis to the TCA cycle. Approximately 94.7% (16,328/17,234) of the predicted core genes were assigned to COG functional categories. The predicted core genes include a relatively high percentage of genes assigned to the following COG categories: translation, ribosomal structure and biogenesis (J), general function prediction only (R), amino acid transport, and metabolism (E), energy production and conversion (C), and unknown function (S) (Figure 2A). Due to a larger fraction of putative or hypothetical genes, only 74.1% (14,622/19,723) of flexible genes were assigned to COG functional categories. Compared with the core genes, flexible genes contain an overrepresentation of genes assigned to the following COG categories: cell motility (N), secondary metabolites biosynthesis, inorganic ion transport and metabolism (P), lipid transport and metabolism (I; Figure 2B). Most of the flexible genes were sourced from the genetic island regions.

Genomic rearrangements and highly variable genetic islands were prevalent features as comparison of gene composition and arrangement in these 14 strains. Integrase and ICE were frequently found in their chromosomes, which seemed to be an important force in shaping their genomic composition and evolutionary divergence.

### Photosynthesis gene cluster

**Figure 3:**
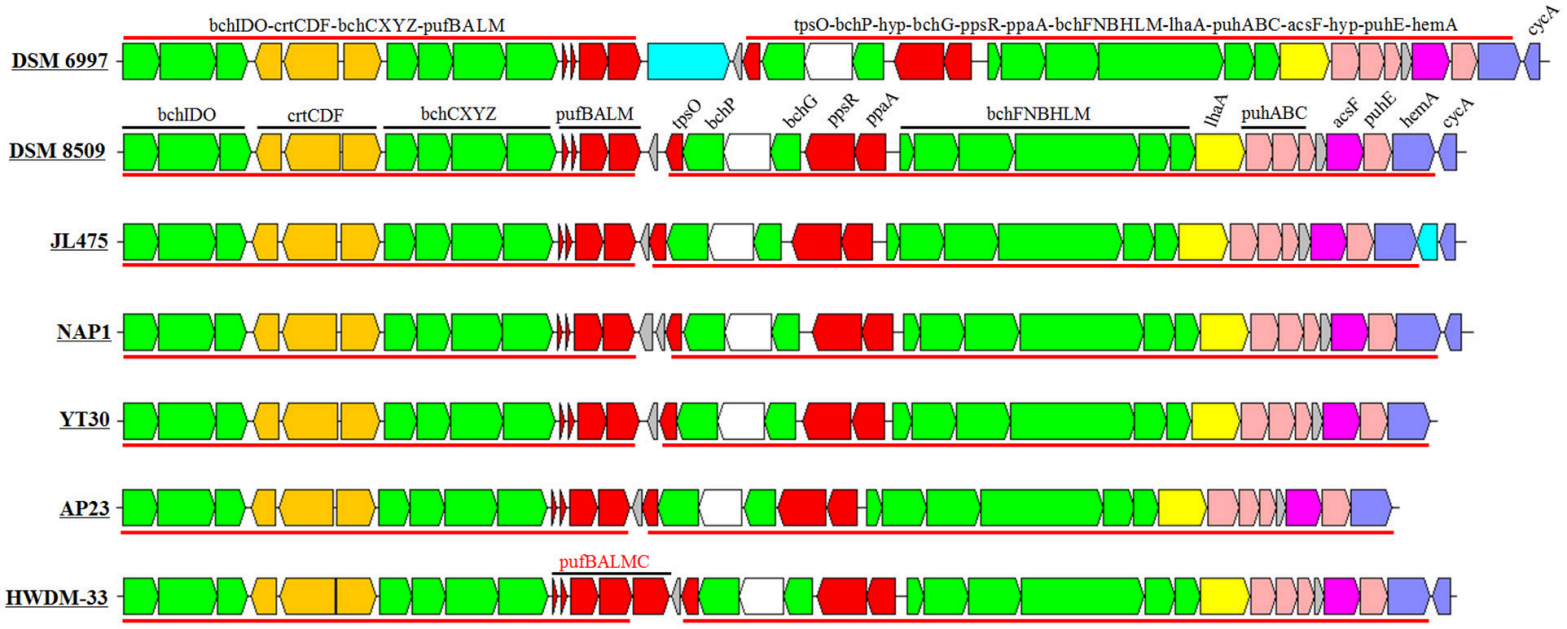
**Structure and arrangement of PGCs in *Erythrobacter***. Green, *bch* genes; red, *puf* and regulator genes; pink, *puh* genes; orange, *crt* genes; blue, *hem* and *cyc* gene; yellow, *lhaA* gene; blank, uncertain or unrelated genes; and gray, hypothetical protein. The horizontal arrows represent putative transcripts.

The sizes of the PGCs ranged from 37.0 to 38.9 kb, which represented ~1.08–1.36% of the genomes. The GC contents of the PGCs varied from 58 to 66%, which was similar to the total GC contents of the corresponding genomes (Table 1). The PGC organization in the *Erythrobacter* genus comprises two conserved subclusters, *bchIDO*-*crtCDF*-*bchCXYZ*-*pufBALM* and *bchFNBHLM*-*lhaA*-*puhABC-acsF-hyp- puhE-hemA* (Figure 3). The PGC arrangement in this genus belonged to type III (forward *crtF-bchCXYZ-puf* plus forward *bchFNBHLM-LhaA-puh*; Zheng et al., 2011, 2013). All PGCs were almost identical in terms of gene arrangement and composition (Figure 3). An inserted gene was involved in the outer membrane protein and the pseudoazurin gene among the PGCs belonging to *E. longus* DSM 6997 and *Erythrobacter* sp. JL475, respectively.

**Figure 4:**
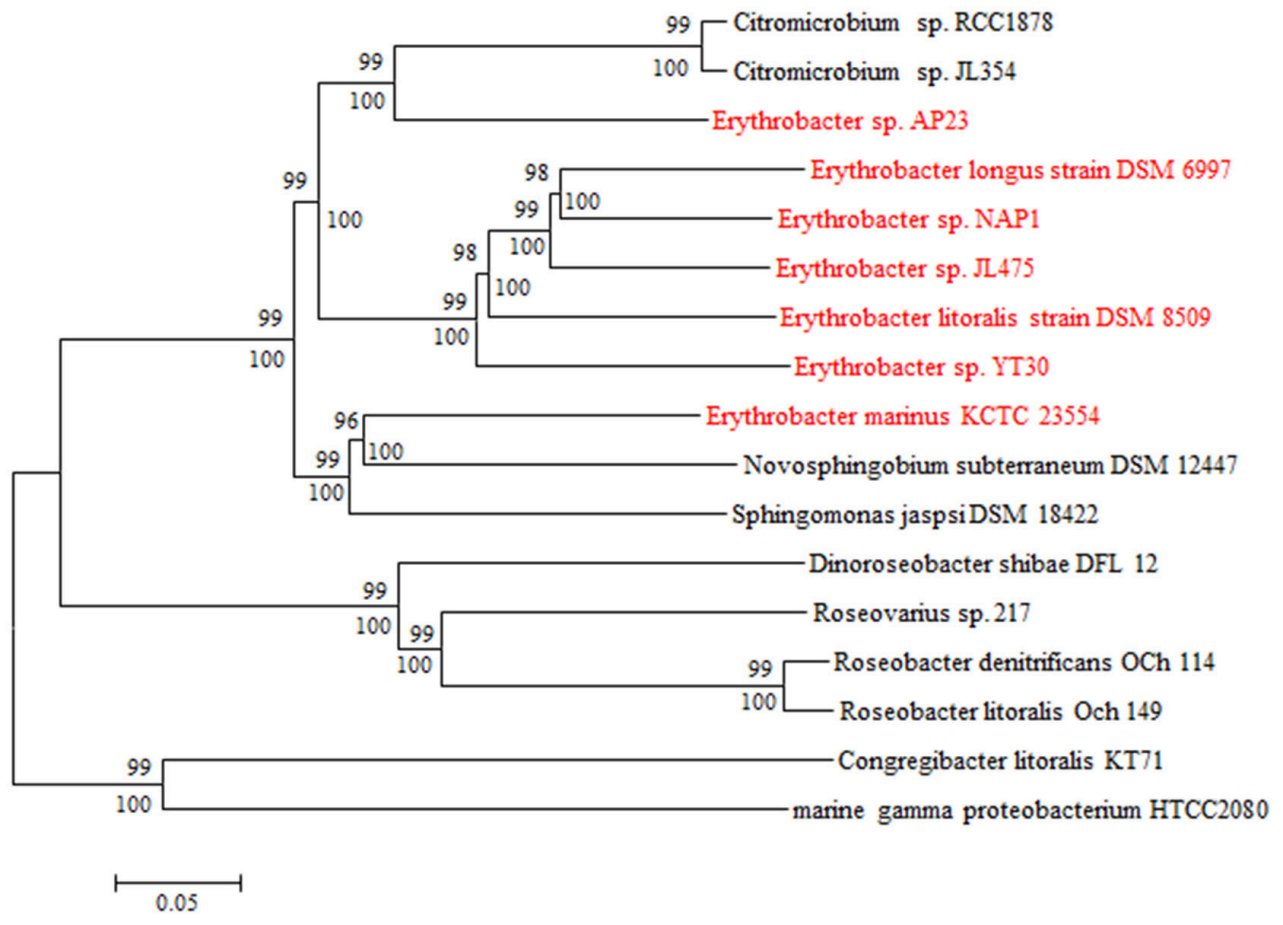
**Neighbor joining phylogenetic analysis of concatenated amino acids sequences of 27 universally conserved genes (9415 positions) in PGCs from GenBank database**. The core genes are *bchBCDFGHILMNOPXYZ-crtCF-pufABLM-lhaA-puhABCE-ascF*. Bar, 0.1 substitutions per amino acids position.

Five AAPB strains belonging to Group I shared the same upstream and the similar downstream genes of PGCs (Table S2), and their PGCs clustered together (Figure 4). This indicated that these five PGCs diverged from a common ancestor. Upstream of these five PGCs, there was a conserved gene cluster in the order of type IV secretion system (T4SS), TonB-dependent transporter and iron ABC transporter. Downstream, the PGCs were flanked by permease, toxin secretion ABC transporter, (outer) membrane protein, and isoquinoline oxidoreductase. The genome of strain HTCC2594 showed genomic recombination close to the corresponding position in the five other Group I AAPB strains. Here, two explanations were proposed to account for the evolution of PGC in Group I: the first is that an HTCC 2594-related strain acquired PGC via horizontal gene transfer a long time ago and then diverged; the other is that some photoheterotrophic ancestors lost the PGC and thus became heterotrophic.

The size of the PGC in strain AP23, which had the highest GC content (66%), was the shortest (37.0 kb) among all known AAPB (Table 1). It had the same gene composition and organization as the AAPB in Group I. However, it displayed a unique flanking gene compared with the AAPB strains in Groups I and III. Integrase, which was inserted after tRNA-Pro-GGG, was found in front of the PGC in strain AP23, and its genes upstream and downstream of PGC were found together in other genomes. The phylogeny indicated that its closest relative was the Citromicrobial strains (Figure 4). Strains LAMA 915 and AP23 shared 99.5% identity at the level of the 16S rRNA gene sequence, and the genome sequence of strain LAMA 915 contained no PGC. That suggested the PGC in strain AP23 was acquired by horizontal gene transfer. In the environment, bacteria are much easier to accept foreign genes from closely related strains (Jain et al., 1999; Koonin et al., 2001). However, it would be difficult to find the HGT event based only on the phylogenetic relationship if that happens.

The PGC in strain HWDM-33 was located after the T4SS. However, there were two copies of T4SS in strains HWDM-33, KA37, SD-21, and YT30, which usually mediates inter-bacterial DNA transfer, and secretion of virulence factors into target cells (Yeo and Waksman, 2004; Alvarez-Martinez and Christie, 2009). The first copy found in all strains coevolved with their genomes based on the phylogeny of the *virB4* gene (Figure S2), and the second copy only detected in four strains (HWDM-33, KA37, SD-21, and YT30) appeared to be obtained by HGT. The type IV secretion system was frequently found in *Alphaproteobacteria*, and it has been predicted to play roles in natural transformation as a mechanism for gene exchange (Hubber et al., 2004; Aylward et al., 2013). In addition, the reaction center (RC) consists of three *pufLMC* genes in strain HWDM-33 instead of *pufLM*, which existed in six other AAPB strains in this genus. Thus, we speculated that the type IV secretion of the T-DNA complex (Table S3) might mediate the HGT of the PGC in strain HWDM-33.

There are two known types of RC in AAPB: one has a tightly bound subunit of a c-type cytochrome (*pufLM-pufC*) that acts as the direct electron donor to a photo-oxidized special pair of bacteriochlorophylls; the other type accepts electrons directly from water-soluble electron carriers such as cytochrome c2 (*cycA*; Nitschke and Dracheva, 1995). In all *Erythrobacter* AAPB genomes except strain HWDM-33, the *pufC* gene was absent. The recent study suggested that the *pufC* gene is not essential for photosynthetic growth and that it might accelerate the re-reduction of the primary electron donor (Verméglio et al., 2012).

A previous study showed that the main difference among PGCs was the genes encoding the carotenoid biosynthetic pathway. The complete set of *crt* genes identified in *Rba. capsulatus* was *crtAIBKCDEFJ* (Zheng et al., 2011). A slightly reduced set of genes was found in some *Roseobacter* and NOR5 species. In comparison, only *crtCDF* existed in the PGCs of the *Erythrobacter* genus. Interestingly, some key genes (*crtYIB, crtWZ*, and *crtG*) for pigment biosynthesis were not organized in the PGCs in *Erythrobacter*, and instead they are scattered in the chromosome. The *crt* genes (*crtYIB* and *crtWZ*) were observed in all seven analyzed non-AAPB strains.

### Integrative and conjugative elements

The genome size of all AAPB except strain HWDM-33 is significantly larger than any of non-AAPB strains. AAPB or non-AAPB containing ICE structures possess relatively large genome size compare to AAPB or non-AAPB without ICE respectively (Table 1). The large pan-genome size of *Erythrobacter* indicated that it had some capacity to obtain foreign genes, and these frequently found ICEs contributed to the flexible genome and possibly to environmental adaptation.

**Figure 5:**
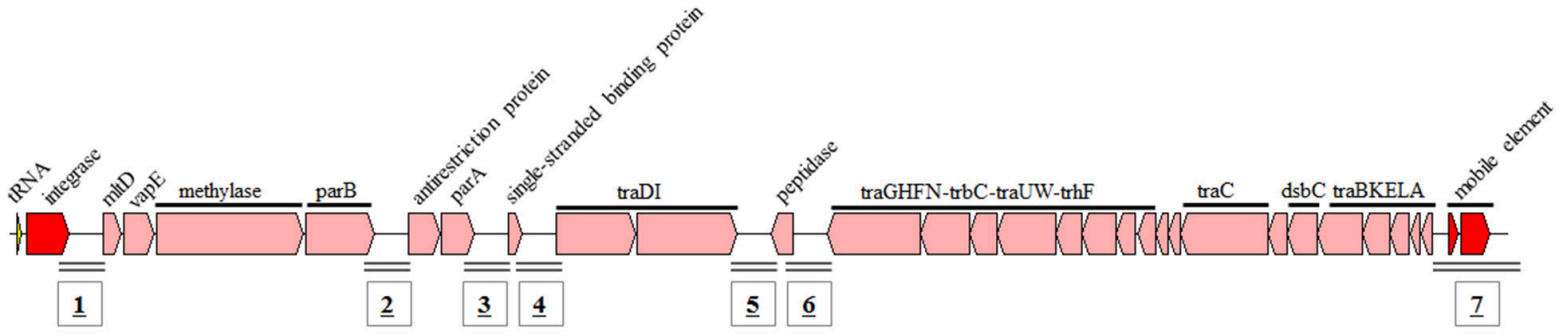
**Structure and composition of ICE**. Seven hotspots (from No. 1 to 7) carrying exogenous genes were detected in the ICEs.

ICEs are bacterial self-transmissible mobile genetic elements that can integrate into and be excised from the chromosome (Böltner et al., 2002; Burrus et al., 2006). ICEs possess features of both temperate bacteriophages (the front part) and conjugative plasmids (the latter part; Figure 5; Burrus et al., 2006; Wozniak et al., 2009). ICEs have recently been shown to contain several intergenic hotspots where a variety of new traits and adaptive functions can be obtained, including resistance to antimicrobial compounds, heavy metals or bacteriophage infection (Wozniak et al., 2009). The core regions display conservative gene synteny with fewer insertions or deletions, which suggests their importance for ICE self-transfer (Gaillard et al., 2010; Miyazaki et al., 2015). ICEs have been reported to be the most abundant conjugative elements in bacteria (Guglielmini et al., 2011; Poulin-Laprade et al., 2015), and they are a major driving force of bacterial genome plasticity and evolution (Böltner et al., 2002; Burrus et al., 2006).

Eight of fourteen genomes were found to contain 14 ICEs. Strain HTCC2594 possessed three ICEs; strains s21-N3, JL475, AP23, and DSM 6997 contained two ICEs; strains SD-21, LAMA915, and NAP1 had one ICE. Two ICEs in strain JL475 were combined together, and one of them lost part of its genes for phage functions. Therefore, there were a total of 13 integrases in the 14 ICEs.

All 14 ICEs, except for the incomplete one in strain JL475, were integrated into the host chromosome after a tRNA gene and could be grouped into three clades based on the different types of tRNA genes. Six ICEs were located after the tRNA-Leu-CAG gene; five ICEs were flanked by the tRNA-Met-CAT gene; three ICEs were integrated after the tRNA-Val-CAC gene.

ICEs identified a specific chromosome position (different tRNA) and then integrated into the chromosome in the 5′–3′ or 3′–5′ direction. The tRNA-Val-CAC gene is always found at the end of the ICEs, indicating that the ICE integrated into the chromosome in the 3′–5′ direction. The other ICEs integrated into the chromosome in the 5′–3′ direction, and the integrase was just after the tRNA-Met-CAT or tRNA-Leu-CAG. This suggested that different types of ICEs underwent site-specific and direction-specific insertions. Interestingly, two integration events occurred at the tRNA-Leu-CAG gene position in strains AP23 and s21-N3.

Two complete prophages were detected in strains KA37 and LAMA915. The prophage in strain KA37 encoded an integrase and was integrated into the host chromosome after the tRNA-Tyr-GTA gene. This integrase was considered a reference and also added into the following analysis.

**Figure 6:**
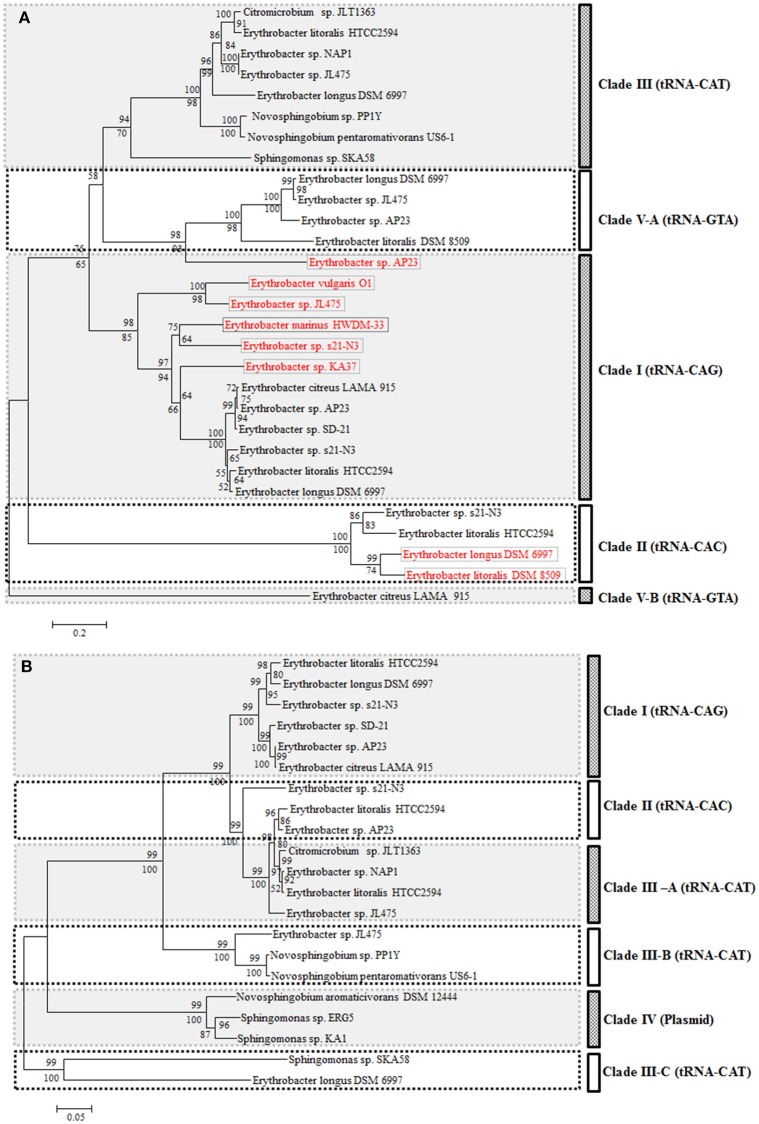
**Neighbor-joining phylogenetic trees based on Integrase (A, 430 amino acid positions) and TraC (B, 846 amino acid positions)**. Bootstrap percentages from both neighbor-joining (above nodes) and maximum likelihood (below nodes) are shown.

All integrases after four tRNAs (tRNA-Leu-CAG, tRNA-Met-CAT, tRNA-Val-CAC, and tRNA-Tyr-GTA) in 14 genomes were collected for the phylogenetic analysis (Figure 6A). Generally, all integrases originating from the same tRNA gene were clustered together except the one in the prophage. These integrases from the ICEs formed three clades (I, II, and III) based on the three tRNA types (Figure 6A). Integrases, which were not from ICEs, also clustered with the corresponding tRNA clades. However, the integrase of the prophage was completely different from those found in other genomes at the tRNA-Tyr-GTA gene position (Figure 6A). This suggested that different types of phages could integrate into the same position in the chromosome.

As seen from the comparison of the phylogeny based on the integrase and TraC, the evolution of the two parts was not completely synchronous. The phylogeny based on the TraC showed more diversity than the integrase. However, these TraC sequences were clustered together and located at the same tRNA position (Figure 6B). Clade III could be grouped into three sub-clades based on the TraC sequences. All of the sequences from the ICEs were distinguished by plasmid origins (Figure 6B).

It seemed that the ICEs possessed incompatibility similar to plasmids. The ICEs from the same host fell into different phylogenetic clades. Two ICEs were located at the same position in strain JL475, but one was an incomplete or defective element. Furthermore, two of them fell into different sub-clades.

From the 14 ICEs, 7 potential hotspots carrying foreign genes were identified (Figure 5) and the inserted gene fragment ranged from 1–2 kb to ~100kb. Overall, 33 and 34 mobile elements were found in 6 and 3 ICEs belonging to Clades I and II, respectively. Only three mobile elements were detected in 5 IECs belonging to Clade III, suggesting that the three types of ICEs displayed different capacities for gene exchange between the ICE and the host genome.

Members of ICEs in different clades were responsible for carrying different functional genes. The ICEs in clade I preferred to carry genes coding for a nucleotide-metabolism-related function, such as a complete type I restriction-modification system, type IIS restriction enzyme, DNA double-strand break repair protein, DEAD-box helicase-related protein, superfamily I/II DNA/RNA helicases, ribonucleotide reductase of class III, DNA methyltransferase, and so on. In addition, a complete respiratory nitrate reductase system was discovered in strain DSM 6997.

Members of the ICEs in clade III mainly carried heavy metal resistance genes, including lead, cadmium, zinc, mercury, nickel, cobalt, and arsenicals. An 18.5-kb DNA fragment involved in heavy metal resistance in the clade III ICE of strain HTCC 2594 (Positions 905,233 to 923,810) was identical to that found in *Citromicrobium* sp. JLT1363 (AEUE01000001, positions 368,467 to 387,044), which also was located in the clade III ICE. These two strains shared 94.8% identity at the level of the 16S rRNA gene sequences, suggesting that the gene exchange mediated by ICE and the active distribution of ICE were ongoing.

The exogenous genes in clade II ICEs were mainly involved in fatty acid metabolism and (outer) membrane proteins, such as receptors, permeases, lipoproteins, phytochromes, and Na^+^/H^+^ antiporters. These different types of ICEs with distinct foreign genes may have provided their own selective benefits under diverse environments to their hosts.

## Conclusion

A comparison of 14 genomes with scattered distribution of AAPB revealed the gain and loss of phototrophic genes co-occurring in the evolutionary history of the genus *Erythrobacter*. The *Erythrobacter* genomes diverged into three separated groups with a large range of genome sizes. The ICEs might play significant roles in shaping or contributing to the large pan-genome of *Erythrobacter*. This study broadens our understanding of the phototrophic lifestyle evolutionary processes. With more novel species identified and whole genomes sequenced in this genus, future detailed analysis should further clarify the evolutionary history of phototrophy.

## Author contributions

Conceived and designed the experiments: QZ, NJ, CC. Performed the experiments: QZ, WL, YL, CC. Analyzed the data: QZ, WL, YL, CC. Contributed reagents/materials/analysis tools: WL, YL. Wrote the paper: QZ, WL, YL, CC, NJ.

### Conflict of interest statement

The authors declare that the research was conducted in the absence of any commercial or financial relationships that could be construed as a potential conflict of interest.

## Supplementary material

Figure S1 **Sizes of the core (green) and pan-genomes (blue) of the Erythrobacter strains**.

Figure S2 **Neighbor-joining phylogenetic trees based on VirB4 amino acid sequences**. Bootstrap percentages from both neighbor-joining (above nodes) and maximum likelihood (below nodes) are shown.

Table S1 **Average Nucleotide Identity by pairwise genome comparison**.

Table S2 **The upstream and downstream genes of PGCs in seven AAPB genoems**.

Table S3 **Annotation for T4SS complex and T-DNA complex**.
