# Supplementary figures and images for: A Comparison of 14 Erythrobacter Genomes Provides Insights into the Genomic Divergence and Scattered Distribution of Phototrophs
Source: Front Microbiol. 2016 Jun 24;7:984. doi: 10.3389/fmicb.2016.00984 (PMC4919336; doi:10.3389/fmicb.2016.00984)

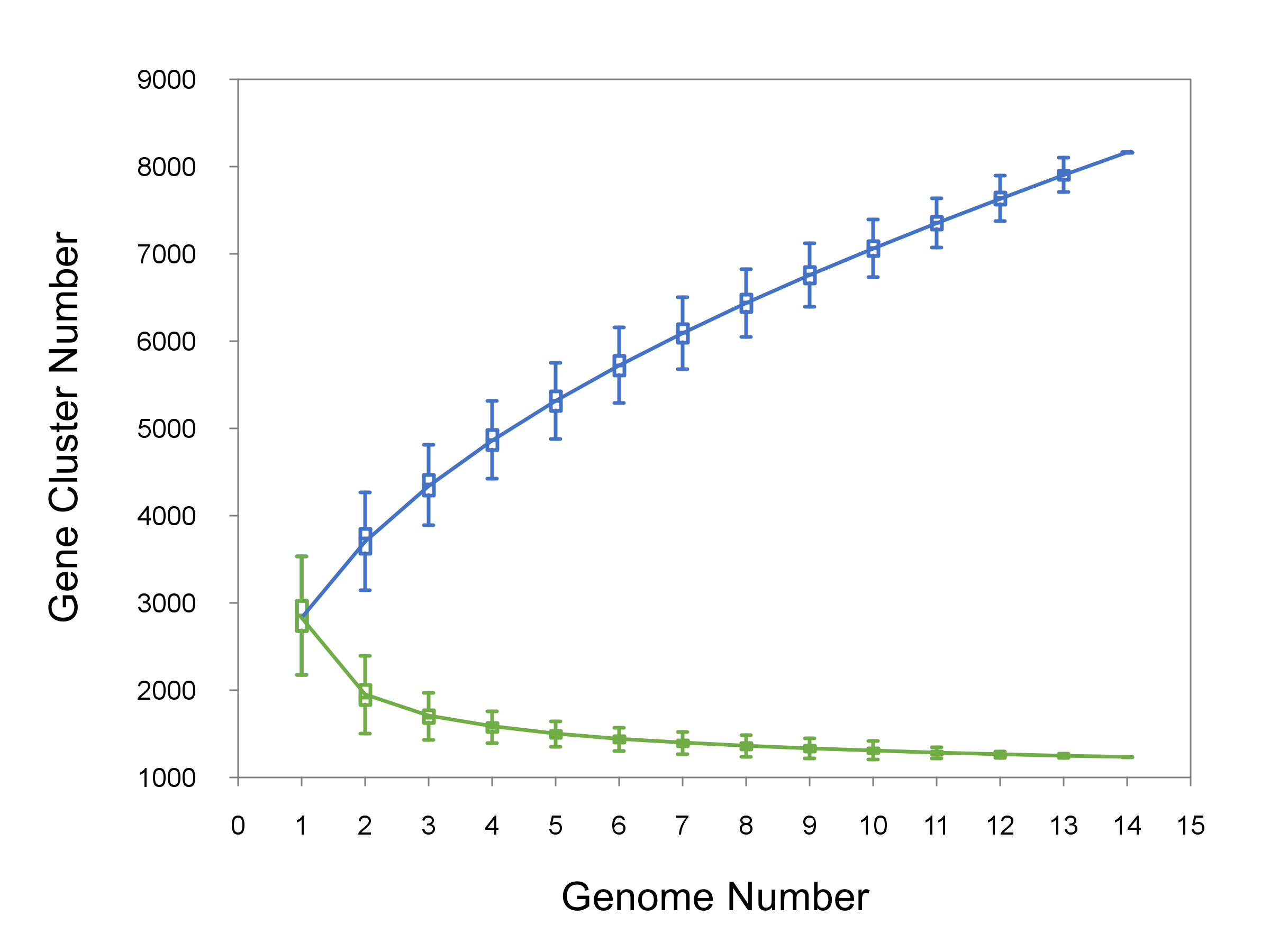

Supplement: Figure S1 — Sizes of the core (green) and pan-genomes (blue) of the Erythrobacter strains. [file Image1.PNG]

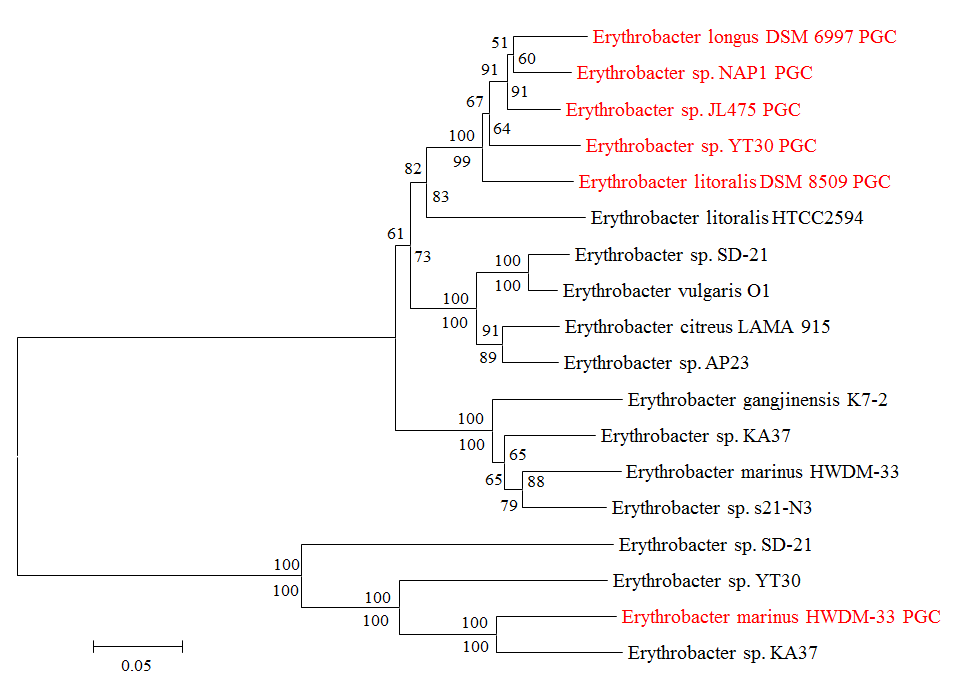

Supplement: Figure S2 — Neighbor-joining phylogenetic trees based on VirB4 amino acid sequences. Bootstrap percentages from both neighbor-joining (above nodes) and maximum likelihood (below nodes) are shown. [file Image2.TIF]
