# Supplementary material for: A Comparison of 14 Erythrobacter Genomes Provides Insights into the Genomic Divergence and Scattered Distribution of Phototrophs
Source: Front Microbiol. 2016 Jun 24;7:984. doi: 10.3389/fmicb.2016.00984 (PMC4919336; doi:10.3389/fmicb.2016.00984)
Supplement: Table S1 — Average Nucleotide Identity by pairwise genome comparison. [file Table1.DOC]

|  | DSM 6997 | NAP1 | JL475 | TY30 | DSM_8509 | HTCC2594 | SD-21 | O1 | LAMA_915 | AP23 | K7-2 | KA37 | s21-N3 | HWDM-33 |
| --- | --- | --- | --- | --- | --- | --- | --- | --- | --- | --- | --- | --- | --- | --- |
| DSM 6997 | * | 73.39  [53.31] | 73.37  [52.28] | 72.07  [45.71] | 72.16  [42.59] | 71.06  [36.97] | 70.93  [33.72] | 70.75  [32.15] | 70.81  [35.02] | 70.78  [36.77] | 69.56  [28.78] | 69.25  [28.67] | 69.41  [29.47] | 69.44  [30.24] |
| NAP1 | 73.50  [59.03] | * | 75.80  [64.51] | 72.68  [53.68] | 74.34  [54.66] | 73.13  [45.70] | 72.43  [42.18] | 72.33  [41.06] | 72.27  [42.76] | 72.44  [46.17] | 70.86  [35.56] | 70.93  [35.64] | 69.80  [34.35] | 69.88  [35.97] |
| JL475 | 73.44  [57.83] | 75.61  [64.98] | * | 73.00  [53.99] | 75.20  [55.61] | 72.92  [45.40] | 72.51  [41.82] | 72.42  [39.67] | 72.63  [42.67] | 72.63  [45.66] | 71.05  [35.01] | 71.42  [35.44] | 70.37  [34.64] | 70.38  [35.74] |
| TY30 | 72.06  [51.97] | 72.73  [54.55] | 73.14  [55.08] | * | 72.30  [53.79] | 70.86  [40.31] | 70.48  [37.41] | 70.70  [37.12] | 70.49  [37.94] | 70.51  [40.86] | 69.65  [34.69] | 69.11  [32.76] | 68.85  [32.51] | 69.23  [34.13] |
| DSM_8509 | 72.06  [46.64] | 74.35  [53.85] | 75.37  [55.08] | 72.39  [51.31] | * | 73.55  [40.66] | 73.40  [39.35] | 73.15  [38.54] | 73.62  [40.72] | 73.42  [43.17] | 71.79  [35.05] | 72.54  [35.16] | 69.90  [33.35] | 70.52  [34.61] |
| HTCC2594 | 71.59  [44.82] | 73.45  [50.82] | 73.22  [50.04] | 70.71  [42.26] | 73.49  [43.88] | * | 74.59  [52.43] | 73.46  [47.09] | 74.90  [54.01] | 75.19  [55.08] | 71.89  [40.84] | 72.12  [41.35] | 71.31  [41.55] | 70.70  [38.64] |
| SD-21 | 71.02  [40.11] | 72.34  [45.73] | 72.73  [45.10] | 70.42  [39.86] | 73.52  [43.23] | 74.42  [51.64] | * | 81.31  [67.80] | 77.66  [60.09] | 77.92  [59.57] | 71.94  [40.21] | 72.57  [42.49] | 70.78  [40.92] | 70.78  [38.17] |
| O1 | 70.68  [40.79] | 72.28  [46.31] | 72.49  [45.65] | 70.63  [41.63] | 73.30  [44.34] | 73.55  [50.81] | 81.41  [70.81] | * | 76.60  [57.28] | 76.76  [59.69] | 72.07  [42.53] | 72.40  [44.46] | 70.35  [39.28] | 70.56  [41.40] |
| LAMA_915 | 70.92  [40.23] | 72.34  [44.96] | 72.90  [44.80] | 70.45  [38.91] | 73.75  [43.03] | 74.78  [51.23] | 77.71  [58.64] | 76.69  [52.48] | * | 81.22  [68.54] | 71.97  [38.28] | 72.70  [41.22] | 71.07  [40.41] | 71.09  [36.96] |
| AP23 | 70.95  [40.96] | 72.31  [45.75] | 72.73  [45.14] | 70.49  [38.36] | 73.37  [41.65] | 74.93  [49.29] | 77.83  [54.28] | 76.83  [50.01] | 80.98  [64.42] | * | 71.81  [36.88] | 72.42  [37.91] | 70.78  [38.19] | 70.54  [37.53] |
| K7-2 | 69.69  [37.45] | 71.02  [41.39] | 71.27  [42.01] | 69.60  [39.75] | 71.88  [41.63] | 71.93  [45.79] | 71.92  [43.84] | 71.98  [43.99] | 72.00  [43.35] | 72.00  [45.08] | * | 74.58  [56.65] | 72.82  [53.41] | 73.47  [57.82] |
| KA37 | 69.40  [33.91] | 71.07  [37.93] | 71.60  [38.36] | 69.26  [34.13] | 72.54  [38.57] | 72.41  [41.97] | 72.83  [41.58] | 72.75  [41.18] | 72.87  [42.13] | 72.84  [41.89] | 74.58  [52.14] | * | 72.81  [50.89] | 73.32  [53.68] |
| s21-N3 | 69.68  [36.31] | 69.60  [38.45] | 70.35  [39.27] | 68.78  [33.60] | 69.69  [36.61] | 70.92  [41.87] | 70.92  [41.25] | 70.16  [37.05] | 70.96  [43.05] | 70.54  [43.48] | 72.77  [48.05] | 72.61  [50.01] | * | 74.30  [56.72] |
| HWDM-33 | 69.37  [38.09] | 69.70  [40.93] | 70.40  [41.32] | 69.13  [38.15] | 70.37  [39.85] | 70.46  [41.51] | 70.59  [40.21] | 70.43  [41.28] | 70.75  [40.82] | 70.42  [44.42] | 73.43  [55.26] | 73.09  [56.26] | 74.33  [59.81] | * |
