# Supplementary material for: A Comparison of 14 Erythrobacter Genomes Provides Insights into the Genomic Divergence and Scattered Distribution of Phototrophs
Source: Front Microbiol. 2016 Jun 24;7:984. doi: 10.3389/fmicb.2016.00984 (PMC4919336; doi:10.3389/fmicb.2016.00984)
Supplement: Table S3 — Annotation for T4SS complex and T-DNA complex. [file Table3.DOC]

**Table S3** Annotation for T4SS complex and T-DNA complex

| Contig | Start | Stop | Length (bp) | Function |
| --- | --- | --- | --- | --- |
| LBHU01000004 | 24430 | 23456 | 975 | ATPase required for both assembly of type IV secretion complex and secretion of T-DNA complex (VirB11) |
| LBHU01000004 | 25480 | 24437 | 1044 | Inner membrane protein forms channel for type IV secretion of T-DNA complex (VirB10) |
| LBHU01000004 | 26193 | 25477 | 717 | Outer membrane and periplasm component of type IV secretion of T-DNA complex, has secretin-like domain (VirB9) |
| LBHU01000004 | 26951 | 26193 | 759 | Inner membrane protein forms channel for type IV secretion of T-DNA complex (VirB8) |
| LBHU01000004 | 28174 | 26948 | 1227 | Inner membrane protein of type IV secretion of T-DNA complex (VirB6) |
| LBHU01000004 | 30546 | 28174 | 2373 | ATPase provides energy for both assembly of type IV secretion complex and secretion of T-DNA complex (VirB4) |
| LBHU01000004 | 30830 | 30543 | 288 | Inner membrane protein forms channel for type IV secretion of T-DNA complex (VirB3) |
